# Broadband perovskite quantum dot spectrometer beyond human visual resolution

**DOI:** 10.1038/s41377-020-0301-4

**Published:** 2020-04-29

**Authors:** Xiaoxiu Zhu, Liheng Bian, Hao Fu, Lingxue Wang, Bingsuo Zou, Qionghai Dai, Jun Zhang, Haizheng Zhong

**Affiliations:** 1 MIIT Key Laboratory for Low-dimensional Quantum Structure and Devices, Beijing Institute of Technology, 100081 Beijing, China; 2 School of Materials Science & Engineering, Beijing Institute of Technology, 100081 Beijing, China; 3 School of Information and Electronics & Advanced Research Institute of Multidisciplinary Science, Beijing Institute of Technology, 100081 Beijing, China; 4 Beijing Key Laboratory of Nanophotonics and Ultrafine Optoelectronic Systems, School of Optics and Photonics, Beijing Institute of Technology, 100081 Beijing, China; 5 Department of Automation & School of Information Science and Technology, Tsinghua University, 100086 Beijing, China; 6 Beijing National Research Center for Information Science and Technology, 100086 Beijing, China

**Keywords:** Quantum dots, Imaging and sensing

## Abstract

The quantum dot spectrometer, fabricated by integrating different quantum dots with an image sensor to reconstruct the target spectrum from spectral-coupled measurements, is an emerging and promising hyperspectrometry technology with high resolution and a compact size. The spectral resolution and spectral range of quantum dot spectrometers have been limited by the spectral variety of the available quantum dots and the robustness of algorithmic reconstruction. Moreover, the spectrometer integration of quantum dots also suffers from inherent photoluminescence emission and poor batch-to-batch repeatability. In this work, we developed nonemissive in situ fabricated MA_3_Bi_2_X_9_ and Cs_2_SnX_6_ (MA = CH_3_NH_3_; *X* = Cl, Br, I) perovskite-quantum-dot-embedded films (PQDFs) with precisely tunable transmittance spectra for quantum dot spectrometer applications. The resulting PQDFs contain in situ fabricated perovskite nanocrystals with homogenous dispersion in a polymeric matrix, giving them advantageous features such as high transmittance efficiency and good batch-to-batch repeatability. By integrating a filter array of 361 kinds of PQDFs with a silicon-based photodetector array, we successfully demonstrated the construction of a perovskite quantum dot spectrometer combined with a compressive-sensing-based total-variation optimization algorithm. A spectral resolution of ~1.6 nm was achieved in the broadband of 250–1000 nm. The performance of the perovskite quantum dot spectrometer is well beyond that of human eyes in terms of both the spectral range and spectral resolution. This advancement will not only pave the way for using quantum dot spectrometers for practical applications but also significantly impact the development of artificial intelligence products, clinical treatment equipment, scientific instruments, etc.

## In situ fabricated perovskite quantum dots put the hyper into spectrometer

A new hyper-spectrometer can “see” more of the electromagnetic spectrum and with greater detail than the human eye., a jointed team led by Prof. Jun Zhang at Beijing Institute of Technology report the fabrication of a “hyper-spectrometer” by integrating an array of 361 different perovskite quantum dots homogeneously distributed in a polymeric film with a charge-coupled device photodetector array. The device was able to measure light of wavelength between 250 and 1000 nm, covering a broad range of the ultraviolet, visible, and infrared parts of the electromagnetic spectrum, at a resolution of around 1.6 nanometers, surpassing the spectral range and resolution of the human eye. The approach extends the function of hyper-spectrometers into the ultraviolet region and provides a practical strategy for fabricating low-cost, compact hyper-spectrometers that could find applications in artificial intelligence products and scientific instruments.

## Introduction

The comparison of cameras with human eyes has been frequently discussed and has motivated the development of advanced detection instruments beyond human visualization^1^. One of the primary goals in artificial intelligence is to expand the light detectivity of cameras in terms of the spectral range, spectral resolution, polarization, and spatial resolution^2–6^. The hyperspectrometer integrates imaging sensors with spectral filters or spectral splitters, providing a promising route toward improving both the spectral resolution and spectral range^7–9^. Because the spectral resolution is mainly determined by the variety of spectral filters and spectral splitters, it has been of great interest to develop novel nanophotonic materials for spectral filter applications^10^, including photonic crystal arrays^11,12^, plasmonic arrays^13–17^, grating arrays^18,19^, and nanowires^20^.

In 2015, the concept of a quantum dot (QD) spectrometer was proposed by Bao and Bawendi^21^. By integrating 195 kinds of CdSe QDs or CdS QDs as spectral filters with a CCD camera, a convenient compact hyperspectrometer was demonstrated to operate in the whole visible range (390–690 nm). Compared with the abovementioned nanophotonic materials, QDs are highly demanded spectral filters, the absorption properties of which can be precisely tuned by varying their size and composition^22–24^. However, the spectral resolution of the QD spectrometer approaches only 3.2 nm, which is still not comparable to the human visual resolution for most visible light^25^. Based on the linear measurement formation model, the spectral resolution of the QD spectrometer correlates with the absorption spectral variety of QDs, and the spectral range is determined by the wavelength response of photodetectors and spectrum-matched QDs. Because silicon-based CCD cameras can respond to ultraviolet (UV), visible (Vis) and near-infrared (NIR) light with wavelengths ranging from 200 to 1000 nm, the desire is to develop precise spectra-controllable, nonemissive and environmentally friendly QDs with matched spectral ranges in the UV–Vis–NIR region, which could offer much flexibility and lead to the development of various spectrometry applications, such as flame observation^26,27^ and mineral detection^28^.

Perovskite quantum dots (PQDs) are emerging as easily processed materials that can be alternated with conventional QDs in photonic and optoelectronic applications due to their superior optical properties and easy fabrication process^29–31^. In particular, the in situ fabricated PQD-embedded films (PQDFs) possess wide spectral tunability, high transparency, and easy processability for device integration^32–34^. These features make them potential alternatives to the previously mentioned CdSe- and CdS-based QDs for spectrometer integration. Considering its utilization in the QD spectrometer, the nonemissive absorption feature is highly demanded, though it has seldom been investigated. In this work, we developed nonemissive lead-free MA_3_Bi_2_X_9_ and Cs_2_SnX_6_ (MA = CH_3_NH_3_; *X* = Cl, Br, I)-based PQDFs with tunable transmittance spectra ranging from 250 nm to 1000 nm and demonstrated their use in constructing a compact hyperspectrometer. Considering the ill-posed underdetermination of the hyperspectral reconstruction model, we developed a compressive-sensing-based total-variation optimization algorithm to enhance the reconstruction quality. As a demonstration, a spectral resolution of ~1.6 nm in a broadband of 250–1000 nm was achieved by integrating a filter array of 361 PQDFs with a CCD camera, which is superior to human visualization in terms of both the spectral resolution and spectral range.

## Results

**Fig. 1:**
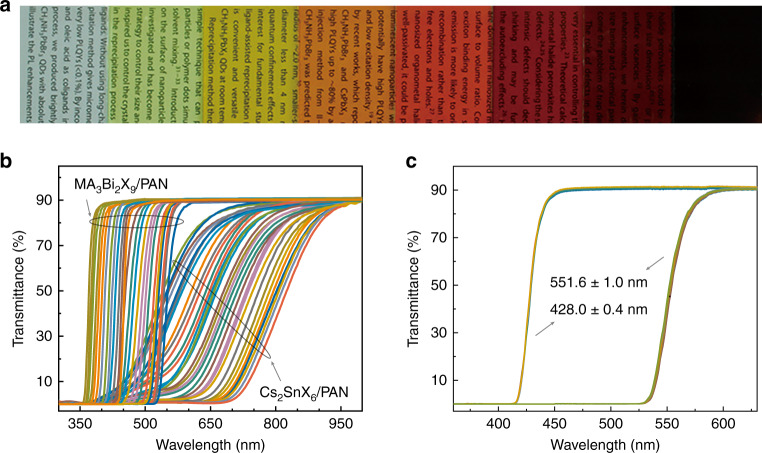
Photographs and transmittance spectra of PQDFs. **a**, **b** Photographs and transmittance spectra for MA_3_Bi_2_X_9_- and Cs_2_SnX_6_-based PQDFs. **c** Transmittance spectra of MA_3_Bi_2_Br_9_/PAN- and MA_3_Bi_2_I_9_/PAN-based PQDFs fabricated from six batches

A large number of spectra-tunable PQDFs were fabricated through in situ fabrication methods. Polyacrylonitrile (PAN) was selected as the polymeric matrix due to its good solubility in *N*,*N*-dimethylformamide (DMF) and good transmittance in the visible and near-infrared regions (Fig. S1a)^35^. MA_3_Bi_2_X_9_ and Cs_2_SnX_6_ were chosen as absorber materials due to their tunable bandgap features^36,37^. The good optical transparency can be well preserved in these as-fabricated PQDFs (Fig. S1b). As shown in Fig. 1a, the as-fabricated MA_3_Bi_2_X_9_/PAN- and Cs_2_SnX_6_/PAN-based PQDFs show tunable colors from colorless to deep red with the conspicuous characteristic of high transparency. As described by Bao’s previous work, the photoluminescence (PL) property is not needed for QD spectrometer integration.^21^ Herein, the PL properties of MA_3_Bi_2_X_9_/PAN- and Cs_2_SnX_6_/PAN-based PQDFs are first investigated. As shown in Fig. S2, these as-fabricated PQDFs are not luminescent under UV-365 nm lamp excitation. The nonemissive feature can be explained by the intrinsic in-direct bandgap and the presence of unpassivated defects on the surface of these in situ fabricated PQDs^38^. Figure 1b shows the transmittance spectra of spectra-tunable MA_3_Bi_2_X_9_/PAN- and Cs_2_SnX_6_/PAN-based PQDFs. By varying the halides, the transmittance spectra can be precisely varied from 250 to 550 nm and 550 to 1000 nm for the MA_3_Bi_2_X_9_/PAN- and Cs_2_SnX_6_/PAN-based PQDFs, respectively. The transmittance of these PQDFs beyond the absorption band edge approaches 90%, implying good transparency. To illustrate the batch-to-batch repeatability, we determined the batch-to-batch variability of the central wavelength at a transmittance of ~45% for two series of samples of MA_3_Bi_2_Br_9_/PAN- and MA_3_Bi_2_I_9_/PAN-based PQDFs with a content of 40 wt.% and a thickness of 20 μm. Figure 1c shows the results of the six batches. The deviation of the central wavelength is within 1 nm, implying the good batch-to-batch reproducibility of the in situ fabrication process.

The optical density (OD), denoted as the signal-to-noise ratio of an optical filter, is usually applied to characterize the light transmittance properties of spectral filters^39^. It can be measured by determining the intensity ratio between the excitation beam and signal beam, usually described by the following equation: OD = *−*log_10_(*T*) (*T* = *I*_*0*_*/I*, where 0 < *T* < 1, where *I*_0_ is the intensity of excitation beam and *I* is the signal beam). The transmittance properties of these as-fabricated PQDFs were determined by using a home-constructed setup, as illustrated in Fig. S3a. A 405 nm wavelength laser beam was used as the incident excitation light source. The excitation beam was incident on the PQDF sample, and the signal beam was received by a spectrometer. To avoid the outrange signal of the spectrometer, neutral-density (ND) filters with a certain OD were used to obtain the intensity of the excitation beam (*I*_0_). To verify the reliability of the method, the OD of commercialized ND filters was measured for comparison (Fig. S3 and Tab. S1). As shown in Fig. S4 and Tab. S1, the ODs of these as-fabricated 20 μm PQDFs are 6.83 at 405 nm for MA_3_Bi_2_(Br_0.8_I_0.2_)_9_/PAN (content of 42 wt.%), 6.70 for MA_3_Bi_2_Br_9_/PAN (content of 40 wt.%) and 6.02 for MA_3_Bi_2_(Br_0.2_I_0.8_)_9_/PAN (content of 39 wt.%). The precisely tunable transmittance spectra and high OD of these PQDFs make them suitable as spectral filters.

**Fig. 2:**
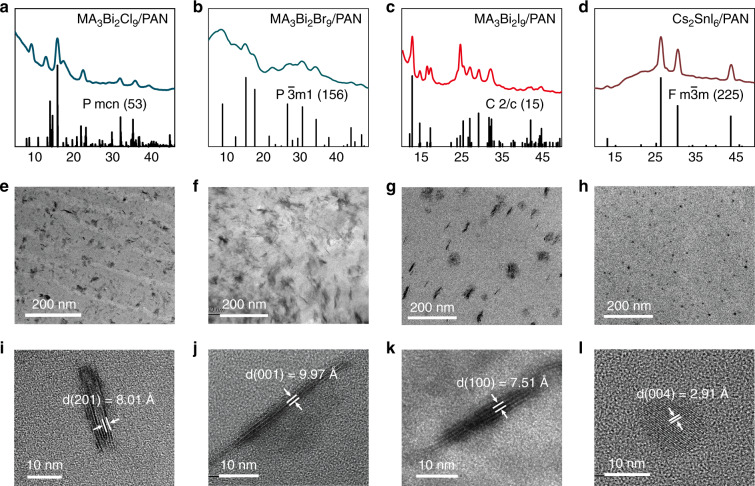
XRD and TEM characterizations of PQDFs. **a**, **e**, **i** XRD patterns, TEM images and HRTEM images for MA_3_Bi_2_Cl_9_/PAN-based PQDFs. **b**, **f**, **j** XRD patterns, TEM images and HRTEM images for MA_3_Bi_2_Br_9_/PAN-based PQDFs. **c**, **g**, **k** XRD patterns, TEM images and HRTEM images for MA_3_Bi_2_I_9_/PAN-based PQDFs. **d**, **h**, **l** XRD patterns, TEM images and HRTEM images for Cs_2_SnI_6_/PAN-based PQDFs

To understand the high transparency of PQDFs, we then characterized them by taking X-ray diffraction (XRD) and transmission electron microscopy (TEM) measurements. As shown in Fig. 2a–d, the XRD patterns of these PQDFs match well with the reference powder XRD files (PDFs)^40^. Figure 2e–i shows typical TEM images of the resulting MA_3_Bi_2_X_9_ and Cs_2_SnX_6_ PQDs in the PAN matrix as well as their corresponding high-resolution TEM (HRTEM) images. The resulting in situ fabricated PQDs are small nanocrystals with a homogeneous dispersion in the PAN matrix, enabling their high transparency. The resulting MA_3_Bi_2_X_9_ nanocrystals are nanoplatelets with a thickness of less than 10 nm for the PQDF with a perovskite content of approximately 40 wt.%, while the resulting Cs_2_SnX_6_ nanocrystals are spherical dots with a diameter of approximately 9.5 nm (Fig. S5). The good crystallinity of these nanocrystals was further confirmed by HRTEM observations. In addition, the observed lattice distances are inconsistent with the expected values for bulk materials.

**Fig. 3:**
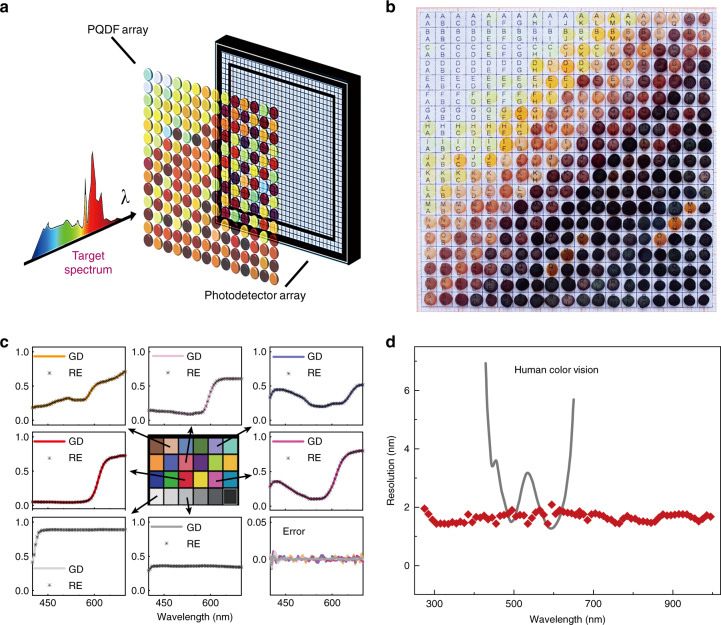
The PQDF-based hyperspectrometer. **a** The architecture of the developed PQDF-based hyperspectrometer. **b** The utilized PQDF filter array, containing 361 different PQDFs and with a size of 7 × 7 cm. **c** The reconstructed spectrum results of a standard X-Rite color checker. GD stands for the ground-truth spectrum, calibrated using a commercial spectrometer, and RE represents the spectrum reconstructed by our method. The bottom-right graph shows the reconstruction error of different swatches. **d** Calibrated spectral resolution of the spectrometer compared with human color vision. The spectral range is 250–1000 nm

To illustrate the potential use of in situ fabricated MA_3_Bi_2_X_9_ and Cs_2_SnX_6_ PQDFs as spectral filters for QD spectrometry, a prototype PQD spectrometer was constructed by integrating a series of PQDF-based filter arrays with a CCD photodetector. The spectral range can be extended to 250 nm at low solid content owing to the strong exciton absorption of Bi-based perovskite (Fig. S6a). As schematically shown in Fig. 3a, b, the key element of the PQD spectrometer is the 7 × 7 cm-sized filter array of 361 kinds of PQDFs with a spectral range 250–1000 nm. Their transmission spectra are shown in Fig. S6b. By expanding the incident light to cover the entire filter, the target spectrum of the incident light is modulated by different PQDFs at different spatial positions. The photodetector array integrates the modulated spectra over the spectrum dimensions. Mathematically, given a spectrum *r*(*λ*) to be measured, the formation model of the sensor can be described by

1 $$b_{i} = {\int_\lambda} {d_{i}\left( \lambda \right)r(\lambda )c(\lambda ){d}\lambda}$$

where *b*_*i*_ is the measurement captured at the corresponding pixel of the sensor, *d*_*i*_(*λ*) is the transmission spectrum of the *i*th PQDF (*i* = 1, 2, 3, …, 361), and *c*(*λ*) is the detection spectrum of the sensor. By discretizing *d*_*i*_(*λ*), *c*(*λ*), and *r*(*λ*) into *d*_*i*_(*k*), *c*(*k*), and *r*(*k*) (*k* = 1, 2, 3, …, *m*), we obtain

2 $$b_{i}^{\prime} = {\sum \limits_{k = 1}^m} {d_{i}(k)c(k)r(k)}$$

$${\rm{Assuming}} \cdot {\boldsymbol{A}} = \left[ {\begin{array}{*{20}{c}} {d_1(1){\mathrm{c}}(1)} & {d_1(2){\mathrm{c}}(2)} & \ldots & {d_1(m){\mathrm{c}}(m)} \\ {d_2(1){\mathrm{c}}(1)} & {d_2(2){\mathrm{c}}(2)} & \ldots & {d_2(m){\mathrm{c}}(m)} \\ \ldots & \ldots & \ldots & \ldots \\ {d_n(1){\mathrm{c}}(1)} & {d_n(2){\mathrm{c}}(2)} & \ldots & {d_n(m){\mathrm{c}}(m)} \end{array}} \right] \in {\boldsymbol{R}}^{n \times m}{\boldsymbol{b}} = [b_1^\prime \,b_2^\prime \ldots \,b_i^\prime ]^T \in R^{n \times m},$$

and ${\boldsymbol{x}} = r(k) \in {\boldsymbol{R}}^{m \times 1}$, the discretized formation model can be written as ***Ax*** = ***b***.

Next, we aim to reconstruct the target spectrum ***x*** from the measurement ***b***. Considering that the measurement formation model is underdetermined, which could degrade the reconstruction quality, using the linear least squares method (refer to Tab. S2 for more details),^21^ we adapted the compressive-sensing (CS)-based total-variation (TV) algorithm^41,42^ for spectral reconstruction, which has been demonstrated to effectively recover latent signals from a small number of measurements. With the TV priori,^43^ the gradient of spectrum ***x*** can be expressed as ***g*** = ***Gx***, where ***G*** is the gradient calculation matrix. Using the *l*_1_ norm to regularize ***g***, the optimization model of the spectrum reconstruction is given by

3 $$\begin{array}{l}\min {|\left| {{\boldsymbol{g}}|} \right|}_{l_1}\\ {\mathrm{s}}.{\mathrm{t}}.\;{\boldsymbol{Gx}} = {\boldsymbol{g}}\\ {\boldsymbol{Ax}} = {\boldsymbol{b}}\end{array}$$

The augmented Lagrangian multiplier (ALM) algorithm was used to solve the model considering its robustness and efficiency^44,45^. The derivations of the algorithm are described in detail in the methods section.

Following the above workflow, we first validated the effectiveness of the PQD spectrometer and calibrated its spectral resolution. Figure 3c shows a comparison between the reconstructed spectra of an X-Rite color checker and their corresponding ground truth. The solid curves are measured relative to the ground truth using a commercial spectrometer (Thorlabs CCS100), and the dotted curves are the reconstructed data. Note that the reconstructed results are consistent with the ground truth. To calibrate the spectral resolution at different wavelengths, we sequentially placed a set of narrow bandpass filters (Thorlabs FL series) in front of the PQD filter, which enables filtering of the incident light with a full width at half maximum (FWHM) of 1 nm. By applying the PQD spectrometer to measure the filtered light, we used the FWHM of the reconstructed spectrum as the calibrated spectral resolution at the corresponding wavelength. Figure 3d shows the calibrated spectral resolution of the PQD spectrometer at different wavelengths ranging from 250 nm to 1000 nm. An average spectral resolution of ~1.6 nm was realized, which is beyond the range of human color vision in the whole visible region^25^. Generally, the spectral resolution can be further increased by increasing the number of QD-based filters. Herein, the spectral resolution of the PQD spectrometer can be further improved by using more PQD filters with well-designed transmittance spectra.

## Discussion

**Fig. 4:**
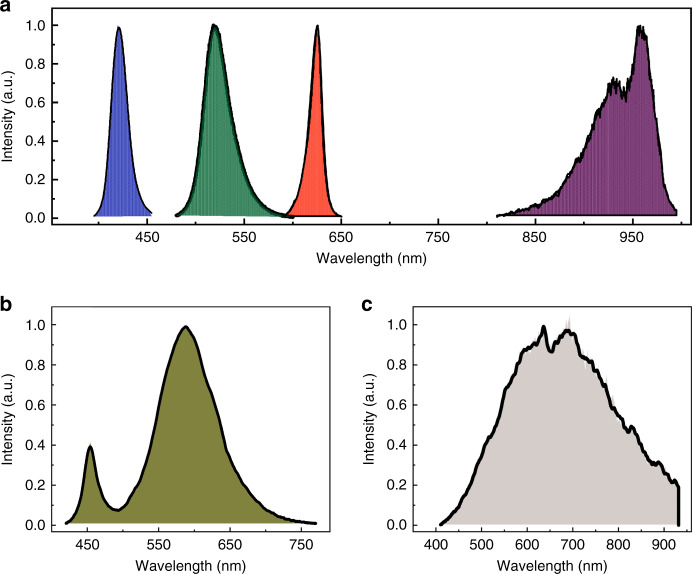
Spectrum measurements of different light sources using the PQDF spectrometer. Spectra reconstruction of **a**, blue, green, red and near-infrared LEDs; (**b**) a white LED; and (**c**) a halogen lamp. The ground-truth spectrum is indicated by the shading, and the black solid curves correspond to our spectrometer measurements

To illustrate the practical applications, the prototype of the PQD spectrometer was further applied to measure the spectra of different commercial light sources, including light-emitting diodes (LEDs) (blue LEDs, green LEDs, red LEDs, NIR LEDs and white LEDs) and halogen lamps. Figure 4 shows the reconstructed spectra from the PQD spectrometer (black solid curves) and the corresponding measured spectra (solid curves with filled shading color) using the commercial spectrometer. We can see that all of the spectra reconstructed at different ranges (including UV, Vis and NIR) match well with the measured spectra. The good agreement implies the potential use of the PQD spectrometer as a broadband and high-resolution measurement instrument in practical applications. In addition, the recent advance in the in situ fabrication of micrometer-sized PQD patterns makes the fabrication of miniature PQD spectrometers possible.

In summary, we have demonstrated the use of spectra-tunable and nonemissive PQDFs to tackle the material challenge for QD spectrometers. Specifically, a number of nonemissive MA_3_Bi_2_X_9_/PAN- and Cs_2_SnX_6_/PAN-based PQDFs with transmittance spectra ranging from 250 to 1000 nm were fabricated by controlling the in situ fabrication progress in the PAN matrix. The as-fabricated PQDF contains homogenous dispersed MA_3_Bi_2_X_9_ and Cs_2_SnX_6_ PQDs in the PAN matrix, which gives them a deep blocking ability at absorbing wavelengths and high transparency at non-absorbing wavelengths. By integrating 361 kinds of PQDFs with a CCD photodetector array, a compact PQD spectrometer with a spectral resolution of ~1.6 nm and spectral range from 250 nm to 1000 nm was achieved using the developed compressive-sensing-based optimization algorithm. The performance of the PQD spectrometer is superior to that of human visualization in terms of both the spectral resolution and spectral range. This advancement provides a practical way to fabricate a low-cost, compact hyperspectrometer with a visual resolution beyond that of humans for sensing applications (Tables S3 and S4). To the best of our knowledge, this work is the first to extend the spectral range of a compact hyperspectrometer into the UV region. It is also expected that the expanded spectral range of the PQD spectrometer will have a great impact on the development of artificial intelligence products, clinical treatment equipment, scientific instruments, etc.

## Methods

### Material fabrication process

The PQD filters were fabricated following the in situ formation process. The fabrication of MA_3_Bi_2_Br_9_/PAN was as follows. A precursor solution was prepared by mixing 72.6 mg MABr, 194 mg BiBr_3_, and 400 mg PAN powder in 3 mL DMF. The precursor solution was dropped onto a smooth glass substrate to form a uniform thickness using the doctor blade method. After 20 min, a uniform and transparent MA_3_Bi_2_Br_9_/PAN-based PQDF was formed. The transmittance spectra of these MA_3_Bi_2_Br_9_/PAN-based PQDFs can be varied by controlling the components, contents and thickness. MA_3_Bi_2_(Cl_*x*_Br_1−*x*_)_9_/PAN and MA_3_Bi_2_(Br_*x*_I_1−*x*_)_9_/PAN were fabricated by varying the halogens following a similar strategy. MACl was dissolved with the addition of HCl at 1 vol.%. The fabrication of Cs_2_SnX_6_/PAN was achieved by the addition of phenethylammonium halide (PEA-X) and 3,3-diphenylpropylamine halide (DPPA-X) following a similar strategy. DPPA-X was prepared by the reaction of DPPA with HBr.

### PQDF characterization

XRD characterizations were analyzed on a Bruker D8 Focus X-ray diffractometer using a Cu Kα radiation source (wavelength of 1.5405 Å). TEM measurements were carried out using a JEOL-JEM 2100 F TEM machine operating at an acceleration voltage of 200 kV. The XRD and TEM results are shown in Fig. 2. Fig. S5 shows the size statistics of the corresponding films. The average diameter of the PQD was 17.9 nm for MA_3_Bi_2_Cl_9_/PAN, 24.8 nm for MA_3_Bi_2_Cl_9_/PAN, 33.1 nm for MA_3_Bi_2_Cl_9_/PAN and 9.5 nm for Cs_2_SnI_6_/PAN. UV–Vis–NIR spectra were recorded using a UV-6100 UV–Vis spectrophotometer (Shanghai Mapada Instruments Co., Ltd., China, range: 200–1000 nm) and a UV-3600 spectrophotometer (Shimadzu, Japan, range: 300–2500 nm). The thickness of the filters was characterized by a surface profiler (Alpha-Step IQ) and ellipsometer (M-2000D).

The OD properties of the PQDFs were measured by using a home-constructed setup, as illustrated in Fig. S3a. It contains a 405 nm wavelength laser beam (Lasever Inc., Ningbo, China), ND filters (Daheng Optics, Beijing, China), a PG2000 high-speed spectrometer (ideaoptics, China, range: 200–1000 nm) and a PQDF. The laser beam was transmitted through the samples, and the signal beam was received by a spectrometer. ND filters with a certain OD were used to obtain the intensity of the excitation beam (*I*_0_). To verify the reliability of the experimental setup, the OD of the ND filters was measured. The OD results for the measurement of the ND filters are shown in Fig. S3b, Fig. S3c and Table S1. The measured ODs for the ND filters of OD1 (0.94), OD2 (2.14) and OD3 (3.06) were not significantly different from those given by the producer, indicating that the experimental setup was accurate and reliable. We used the setup to measure the OD of selected PQDFs with high solid content and a thickness of 20 μm, and the results are shown in Fig. S4 and Table S1. The OD of a PQDF can be up to 6.83 at 405 nm owing to the high absorption coefficient of perovskite. The inset of Fig. S4b shows the weak luminescence under intense excitation light, while nonemission was observed under the same excitation light, indicating the poor luminescence of Bi-based PQD.

### Spectrometer measurements

(i) *QD spectral calibration.* The experimental setup for the QD spectral calibration is shown in Fig. S7. The source light was concentrated into a dot light source by the two diaphragms. The size of the dot light was similar to the size of the dot in the printed film. In the experiments, the light source for calibration was a halogen lamp (Thorlabs SL301, range: 360–3800 nm), and the spectrometer we used was a fiber spectrometer (Thorlabs CCS200/M). By shifting the translation stage, the spectrometer swept the whole printed film dot by dot and measured the corresponding transmitted spectrum of each QD. The calibrated transmission spectrum of the QD was obtained by dividing the measured transmitted spectrum by the spectrum of the light source. In the experiments, we calibrated only the spectrum ranging from 400 to 950 nm. The transmittance spectra of the filter array of PQDFs are shown in Fig. S8.

(ii) *Light source spectral reconstruction*. The light source spectrum reconstruction was similar to the QD spectral calibration. First, we used the spectrometer to measure the transmitted spectrum dot by dot by shifting the translation stage. Then, we integrated each measured transmitted spectrum with respect to the wavelength and acquired the corresponding coupled measurements. Finally, the light source spectra were reconstructed by the derived algorithm based on compressive sensing. The light sources we used in the experiments included blue LEDs, green LEDs, red LEDs, near-infrared LEDs, white LEDs and tungsten lamps.

### Algorithm derivation

The ALM method was utilized to solve the model in Eq. (3). By introducing a Lagrangian multiplier ***y*** to incorporate the equality constraints into the objective function, we obtain the following Lagrangian function:

s1 $$\begin{array}{l}\min L = \|{\boldsymbol{g}}\|_{l_1} + \langle{\boldsymbol{y}}_1,{\boldsymbol{Gx}} - {\boldsymbol{g}}\rangle + \frac{{\mu _1}}{2}\left\|{\boldsymbol{Gx}} - {\boldsymbol{g}}\right\|_{l_2}^2\\ + \langle{\boldsymbol{y}}_2,{\boldsymbol{Ax}} - {\boldsymbol{b}}\rangle + \frac{{\mu _2}}{2}\left\|{\boldsymbol{Ax}} - {\boldsymbol{b}}\right\|_{l_2}^2\end{array}$$

where $\left\langle \cdot \right\rangle$ is the inner product and *μ*_1_ and *μ*_*2*_ are the parameters used to balance different optimization items. This equation can be rewritten as

s2 $$\min L = \Vert{\boldsymbol{g}}\Vert_{l_1} + \frac{{\mu _1}}{2}\left\|{\boldsymbol{Gx}} - {\boldsymbol{g}} + \frac{{{\boldsymbol{y}}_1}}{{\mu _1}}\right\|_{l_2}^2 + \frac{{\mu _2}}{2}\left\|{\boldsymbol{Ax}} - {\boldsymbol{b}} + \frac{{{\boldsymbol{y}}_2}}{{\mu _2}}\right\|_{l_2}^2$$

Following the iterative reconstruction scheme of the ALM, the updating principle of each variable is to minimize the Lagrangian function while keeping the other variables constant. The detailed derivations are as follows:

**Optimize** ***g***. Removing the items irrelevant to ***g***, the objective function becomes

s3 $$\min L({\boldsymbol{g}}) = \Vert{\boldsymbol{g}}\Vert_{l_1} + \frac{{\mu _1}}{2}\left\|{\boldsymbol{Gx}} - {\boldsymbol{g}} + \frac{{{\boldsymbol{y}}_1}}{{\mu _1}}\right\|_{l_2}^2$$

According to the ALM derivation, the updating rule of ***g*** is

s4 $${\boldsymbol{g}} = T_{\frac{1}{{\mu _1}}}\left( {{\boldsymbol{Gx}} + \frac{{{\boldsymbol{y}}_1}}{{\mu _1}}} \right)$$

where $T_{\frac{1}{{\mu _1}}}\left( \cdot \right)$ is the thsholding operator, defined as

s5 $$T_{\frac{1}{{\mu _1}}}\left( x \right) = \left\{ {\begin{array}{*{20}{l}} {x - \frac{1}{{\mu _1}},\,x > \frac{1}{{\mu _1}}} \\ {x + \frac{1}{{\mu _1}},\,x < - \frac{1}{{\mu _1}}} \\ {0,{\,\rm{others}}} \end{array}} \right.$$

**Optimize** ***x***. Removing the items irrelevant to ***x***, the objective function becomes

s6 $$\min L({\boldsymbol{x}}) = \frac{{\mu _1}}{2}\left\| {{\boldsymbol{Gx}} - {\boldsymbol{g}} + \frac{{{\boldsymbol{y}}_1}}{{\mu _1}}} \right\|_{l_2}^2 +\, \frac{{\mu _2}}{2}\left\| {{\boldsymbol{Ax}} - {\boldsymbol{b}} + \frac{{{\boldsymbol{y}}_2}}{{\mu _2}}} \right\|_{l_2}^2$$

and the gradient is

s7 $$\frac{{\partial L({\boldsymbol{x}})}}{{\partial {\boldsymbol{x}}}} = \mu _1{\boldsymbol{G}}^{T}\left( {{\boldsymbol{Gx}} - {\boldsymbol{g}} + \frac{{{\boldsymbol{y}}_1}}{{\mu _1}}} \right) + \mu _2{\boldsymbol{A}}^{T}\left({\boldsymbol{Ax}} - {\boldsymbol{b}} + \frac{{{\boldsymbol{y}}_2}}{{\mu _2}}\right)$$

Setting $\frac{{\partial L({\boldsymbol{x}})}}{{\partial {\boldsymbol{x}}}} = 0$, the closed-form solution of ***x*** is

s8 $${\boldsymbol{x}} = \left( {\mu _1{\boldsymbol{G}}^{T}{\boldsymbol{G}} + \mu _2{\boldsymbol{A}}^{T}{\boldsymbol{A}}} \right)^{ - 1}\left[ {\left( {\mu _1{\boldsymbol{G}}^{T}\left( {{\boldsymbol{g}} - \frac{{{\boldsymbol{y}}_1}}{{\mu _1}}} \right)} \right. + \mu _2{\boldsymbol{A}}^{T}\left( {{\boldsymbol{b}} - \frac{{{\boldsymbol{y}}_2}}{{\mu _2}}} \right)} \right]$$

**Optimize** ***y*** **and** ***μ***. Following the ALM derivation, the Lagrangian multiplier ***y*** and weight parameter *μ* are updated as

s9 $$\begin{array}{l}{\boldsymbol{y}}_1^\prime = {\boldsymbol{y}}_1 + \mu _1({\boldsymbol{Gx}} - {\boldsymbol{g}})\\ {\boldsymbol{y}}_2^\prime = {\boldsymbol{y}}_2 + \mu _2({\boldsymbol{Ax}} - {\boldsymbol{b}})\\ \mu _1^\prime = {\mathrm{min}}(\rho \mu _1,\mu _{1{\rm{max}}})\\ \mu _2^\prime = {\mathrm{min}}(\rho \mu _2,\mu _{2{\rm{max}}})\end{array}$$

where *ρ*, *μ*_1max_, and *μ*_2max_ are set by the users to adjust the growing speed and maximum of *μ*.

Based on the above derivations, the spectral reconstruction algorithm is as follows.

Algorithm for spectral reconstruction

**Input**: measurement vector ***b***, sampling matrix ***A***, gradient calculation matrix ***G***;

algorithm parameters *ρ*, *μ*_1max_, *μ*_2max**;**_

Initialization ***y***_1_ = 0, ***y***_2_ = 0;

**while** *not converged* **do**

1. update ***g*** according to Eq. (s4);
2. update ***x*** according to Eq. (s8);
3. update ***y***_1_, ***y***_2_, *μ*_1_, *μ*_2_ according to Eqs. (s9);

end

**Output**: target spectrum ***x***;

## Supplementary information

Supplementary information

Confidential Certificate

## References

[1] Bacon CP, Mattley Y, DeFrece R (2004). Miniature spectroscopic instrumentation: applications to biology and chemistry. Rev. Sci. Instrum..

[2] Li QT (2019). Transparent multispectral photodetectors mimicking the human visual system. Nat. Commun..

[3] Khorasaninejad M (2016). Metalenses at visible wavelengths: diffraction-limited focusing and subwavelength resolution imaging. Science.

[4] Yakunin S (2017). Non-dissipative internal optical filtering with solution-grown perovskite single crystals for full-colour imaging. *NPG Asia*. Materials.

[5] Goossens S (2017). Broadband image sensor array based on graphene–CMOS integration. Nat. Photonics.

[6] Park, H. L.et al. Retina-inspired carbon nitride-based photonic synapses for selective detection of UV light. *Adv. Mater.*10.1002/adma.201906899 (2020).. 10.1002/adma.201906899 31984573

[7] Fischer C, Kakoulli I (2006). Multispectral and hyperspectral imaging technologies in conservation: current research and potential applications. Stud. Conserv..

[8] Khan MJ (2018). Modern trends in hyperspectral image analysis: a review. IEEE Access.

[9] McGonigle AJS (2018). Smartphone spectrometers. Sensors.

[10] Chen Q (2016). Nanophotonic image sensors. Small.

[11] Kim SH (2010). Integration of colloidal photonic crystals toward miniaturized spectrometers. Adv. Mater..

[12] Bryan KM (2013). Inexpensive photonic crystal spectrometer for colorimetric sensing applications. Opt. Express.

[13] Xu T (2010). Plasmonic nanoresonators for high-resolution colour filtering and spectral imaging. Nat. Commun..

[14] Yokogawa S, Burgos SP, Atwater HA (2012). Plasmonic color filters for CMOS image sensor applications. Nano Lett..

[15] Zheng BY (2014). Color-selective and CMOS-compatible photodetection based on aluminum plasmonics. Adv. Mater..

[16] Jang WY (2016). Experimental demonstration of adaptive infrared multispectral imaging using plasmonic filter array. Sci. Rep..

[17] Duempelmann L, Gallinet B, Novotny L (2017). Multispectral imaging with tunable plasmonic filters. ACS Photonics.

[18] Kanamori Y, Shimono M, Hane K (2006). Fabrication of transmission color filters using silicon subwavelength gratings on quartz substrates. IEEE Photonics Technol. Lett..

[19] Horie Y (2017). Visible wavelength color filters using dielectric subwavelength gratings for backside-illuminated CMOS image sensor technologies. Nano Lett..

[20] Yang ZY (2019). Single-nanowire spectrometers. Science.

[21] Bao J, Bawendi MG (2015). A colloidal quantum dot spectrometer. Nature.

[22] Khan SA, Ellerbee Bowden AK (2016). Colloidal quantum dots for cost-effective, miniaturized, and simple spectrometers. Clin. Chem..

[23] Shirasaki Y (2013). Emergence of colloidal quantum-dot light-emitting technologies. Nat. Photonics.

[24] Saran R, Curry RJ (2016). Lead sulphide nanocrystal photodetector technologies. Nat. Photonics.

[25] Wright WD, Pitt FHG (1934). Hue-discrimination in normal colour-vision. Proc. Phys. Soc..

[26] Glover JH (1975). Chemiluminescence in gas analysis and flame-emission spectrometry. A review. Analyst.

[27] Winefordner JD, Vickers TJ (1972). Flame spectrometry. Anal. Chem..

[28] Zawisza B (2011). Determination of rare earth elements by spectroscopic techniques: a review. J. Anal. At. Spectrom..

[29] Protesescu L (2015). Nanocrystals of cesium lead halide perovskites (CsPbX_3_, X = Cl, Br and I): novel optoelectronic materials showing bright emission with wide color gamut. Nano Lett..

[30] Zhang F (2015). Brightly luminescent and color-tunable colloidal CH_3_NH_3_PbX_3_ (X = Br, I, Cl) quantum dots: potential alternatives for display technology. ACS Nano.

[31] Song JZ (2015). Quantum dot light‐emitting diodes based on inorganic perovskite cesium lead halides (CsPbX_3_). Adv. Mater..

[32] Zhou QC (2016). In situ fabrication of halide perovskite nanocrystal-embedded polymer composite films with enhanced photoluminescence for display backlights. Adv. Mater..

[33] Wang YN (2016). Ultrastable, highly luminescent organic–inorganic perovskite–polymer composite films. Adv. Mater..

[34] Chang S, Bai ZL, Zhong HZ (2018). In situ fabricated perovskite nanocrystals: a revolution in optical materials. Adv. Opt. Mater..

[35] Jung B (2005). Effect of crystallization and annealing on polyacrylonitrile membranes for ultrafiltration. J. Membr. Sci..

[36] Leng MY (2016). Lead-free, blue emitting bismuth halide perovskite quantum dots. Angew. Chem. Int. Ed..

[37] Lee B (2017). Solution processing of air-stable molecular semiconducting iodosalts, Cs_2_SnI_6-*x*_Br_*x*_, for potential solar cell applications. Sustain. Energy Fuels.

[38] Saparov B (2016). Thin-film deposition and characterization of a Sn-deficient perovskite derivative Cs_2_SnI_6_. Chem. Mater..

[39] Southwell WH, Hall RL (1989). Rugate filter sidelobe suppression using quintic and rugated quintic matching layers. Appl. Opt..

[40] Hoye RLZ (2016). Methylammonium bismuth iodide as a lead-free, stable hybrid organic–inorganic solar absorber. Chem.—A Eur. J..

[41] Donoho DL (2006). Compressed sensing. IEEE Trans. Inf. Theory.

[42] Candès EJ, Romberg JK, Tao T (2006). Stable signal recovery from incomplete and inaccurate measurements. Commun. Pure Appl. Math..

[43] Candès EJ, Wakin MB (2008). An introduction to compressive sampling. IEEE Signal Process. Mag..

[44] Lin, Z. C., Liu, R. S. & Su, Z. X. Linearized alternating direction method with adaptive penalty for low-rank representation. In *Proc. Advances in Neural Information Processing Systems* 24 (eds Shawe-Taylor, J., Zemel, R. S., Bartlett, P. L., Pereira, F. & Weinberger, K. Q.) 612–620 (Curran Associates Inc., Red Hook, 2011).

[45] Suo JL (2014). Joint non-Gaussian denoising and superresolving of raw high frame rate videos. IEEE Trans. Image Process..

