# Supplementary material for: Broadband perovskite quantum dot spectrometer beyond human visual resolution
Source: Light Sci Appl. 2020 Apr 29;9:73. doi: 10.1038/s41377-020-0301-4 (PMC7190644; doi:10.1038/s41377-020-0301-4)

**Supplemental Information for** **Broadband perovskite quantum dot spectrometer beyond human visual resolution**

Xiaoxiu Zhu^1,2^, Liheng Bian^1,3*^, Hao Fu^1,3^, Lingxue Wang^4^, Bingsuo Zou^2^, Qionghai Dai^5,6^, Jun Zhang^3^ and Haizheng Zhong^1,2*^

^1^MIIT Key Laboratory for Low-dimensional Quantum Structure and Devices, Beijing Institute of Technology, 100081 Beijing, China.

^2^School of Materials Sciences & Engineering, Beijing Institute of Technology, 100081 Beijing, China.

^3^School of Information and Electronics & Advanced Research Institute of Multidisciplinary Science, Beijing Institute of Technology, 100081 Beijing, China.

^4^Beijing Key Laboratory of Nanophotonics and Ultrafine Optoelectronic Systems, School of Optics and Photonics, Beijing Institute of Technology, 100081 Beijing, China.

^5^Department of Automation & School of Information Science and Technology, Tsinghua University, 100086 Beijing, China.

^6^Beijing National Research Center for Information Science and Technology, 100086 Beijing, China.

**Tab. S1. The result of OD measurement for ND filters using the setup in Fig. S3a.**

| **Samples** | **Data of this paper** | | **Data given by producer** |
| --- | --- | --- | --- |
| MA_3_Bi_2_Br_9_/PAN (40%-20 μm) | 6.70 | - | |
| MA_3_Bi_2_(Br_0.8_I_0.2_)_9_/PAN (42%-20 μm) | 6.83 | - | |
| MA_3_Bi_2_(Br_0.2_I_0.8_)_9_/PAN (39%-20 μm) | 6.02 | - | |
| ND1 (OD1) | 0.94 | 0.93 | |
| ND2 (OD2) | 2.14 | 2.10 | |
| ND3 (OD3) | 3.06 | 3.00 | |

**Tab. S2. Comparison of spectral reconstructions between** the **TV algorithm and the least squares (LS) method at different noise variances.**


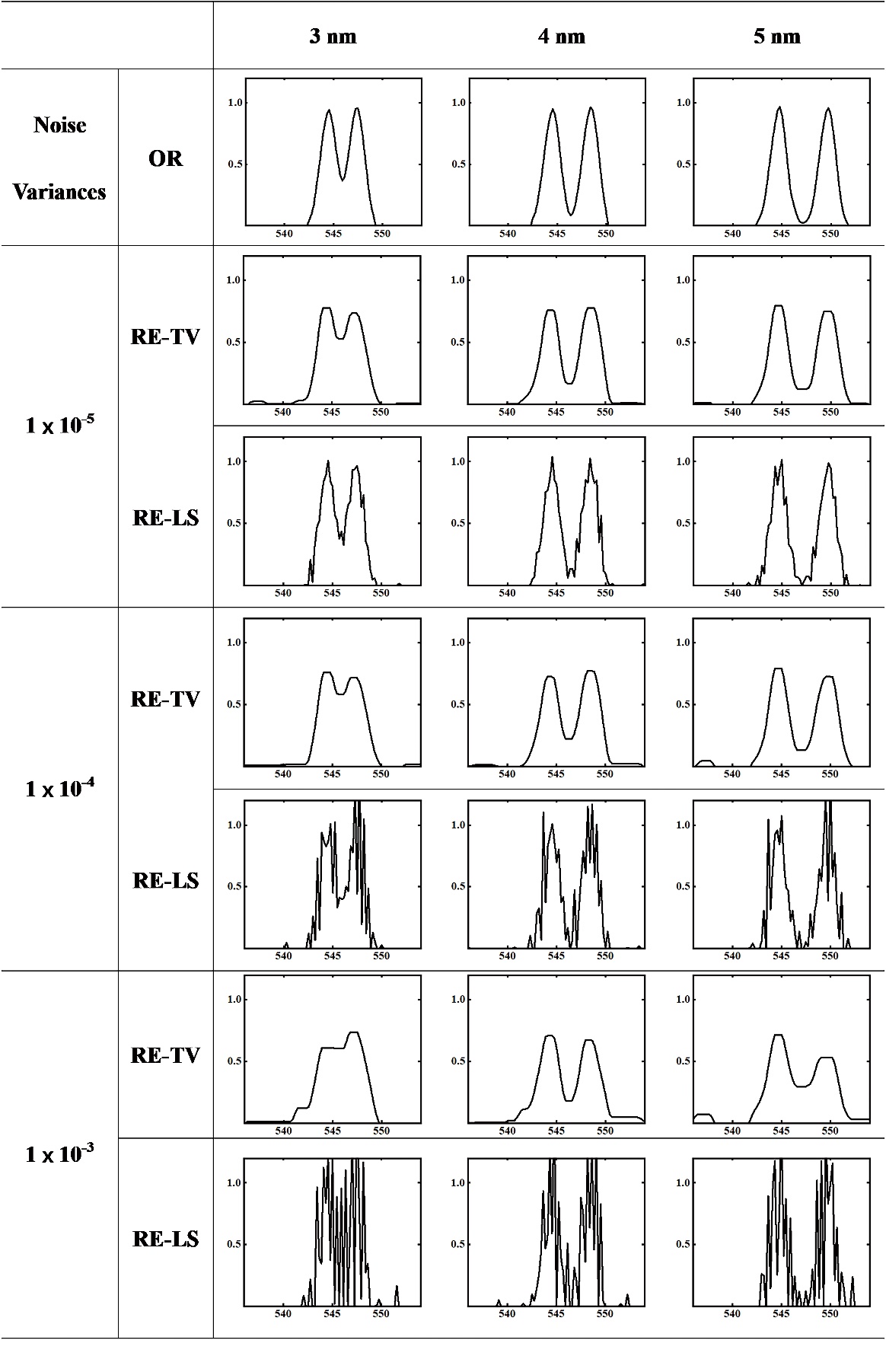


**Tab. S3. Comparison between CdSe QDs and lead-free PQDFs.**

| **Application Requirements** | **CdSe (CdS)** | **Lead-free PQDF** |
| --- | --- | --- |
| Spectral Tunable | √ | √ |
| **Nonemissive** | × (quencher) | **√ (nonemissive)** |
| **Fabrication** | × (hot injection, ex situ) | **√ (in situ)** |
| **Repeatable** | √ | √ |
| Printable | √ | √ |
| **Environmentally Friendly** | × | **√** |

**Tab. S4. Comparison of hyperspectral images between reported work and this work.**

| Sensor | Spectral Range (nm) | Spectral Resolution (nm) | Reference |
| --- | --- | --- | --- |
| Landsat-8 | 450~1250 | 8 | Khan, M. J., Khan, H. S., Yousaf, A., Khurshid, K. & Abbas, A. *IEEE Access* **6,** 14118-14129 (2018). |
| ASTER | 520~11650 | 15~90 |  |
| Hyperion | 400~2500 | 30 |  |
| ALOS | 520~770 | 2.5 |  |
| AVIRIS | 380~2500 | 4~20 |  |
| HyMap | 450~2480 | 2~10 |  |
| ROSIS | 420~8730 | 2 |  |
| DAIS-7915 | 450~12000 | 3~10 |  |
| AISA | 450~900 | 2.9 |  |
| CASI | 430~870 | 2 |  |
| Vidicon PbO-PbS | 400~2200 | 10 | Fischer, C. & Kakoulli, I. *Stud. Conserv.* **51,** 3-16 (2006). |
| CCD | 400~2500 | - |  |
| CCD | 390~690 | 3.2 | Bao, J. & Bawendi, M. G. *Nature* **523,** 67-70 (2015). |
| CCD | **250~1000** | **1.6** | **This Work** |

**Fig. S1. Transmittance spectra of PAN and PQDF in the range from 250 nm to 2500 nm.** **a,** Transmittance and absorption spectra of PAN with a thickness of 24 μm. PAN has little absorption and high transmittance up to 92% in the range of 250~2500 nm. **b,** Transmittance spectra of MA_3_Bi_2_X_9_/PAN-based PQDFs with a thickness of 20 μm and content of 15 wt%. The transmittance in the range of 250~2500 nm can be up to 92%.


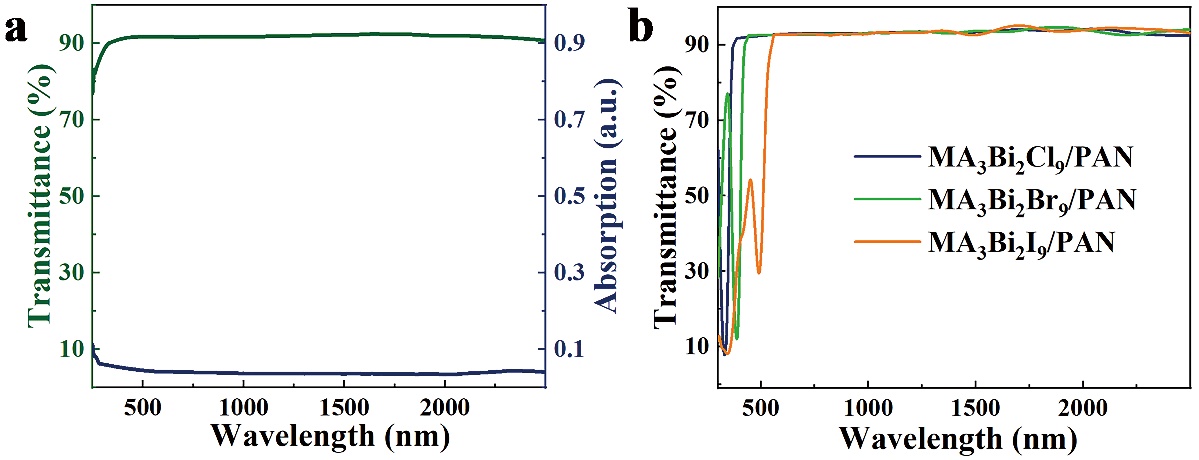


**Fig. S2. Photograph of selected PQDFs under a, natural light and b, UV-254 nm lamp excitation.**


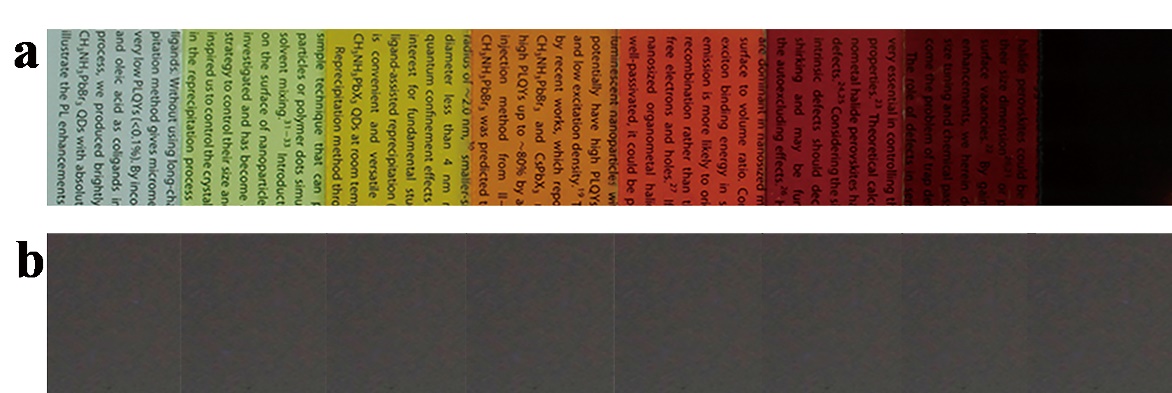


**Fig. S3. OD experimental setup and experimental data of ND filters. a,** Experimental setup. **b,** Experimental data for three kinds of ND filter combinations, i.e., “OD1+OD2+OD3”, “OD2+OD3” and “OD1+OD3”, at the same laser intensity. The y-axis is a single beam measured by the spectrometer. **c,** Experimental data for “OD1+OD2” and OD3 at the same laser intensity. Both have similar counts, indicating the good reliability of the setup.


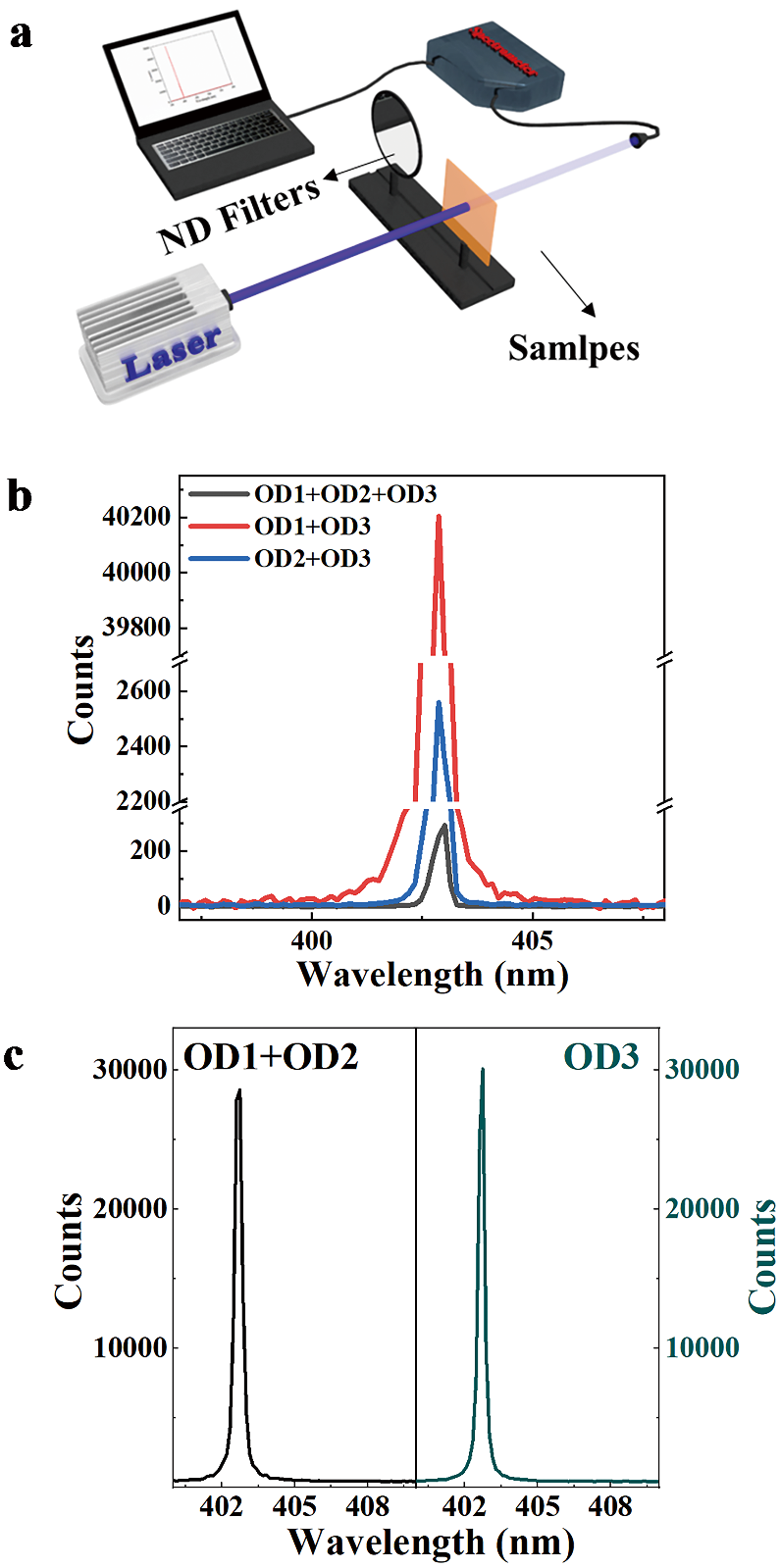


**Fig. S4. Experimental data of representative 20 μm PQDFs using the OD experimental setup.** The left panel is the intensity of the excitation beam, and the right panel is the intensity of the signal beam. **a,** OD 6.83 for MA_3_Bi_2_(Br_0.8_I_0.2_)_9_/PAN (content of 42 wt.%); **b,** OD 6.70 for MA_3_Bi_2_Br_9_/PAN (content of 40 wt.%), with the insert showing weak luminescence under intense excitation light; and **c,** OD 6.02 for MA_3_Bi_2_(Br_0.2_I_0.8_)_9_/PAN (content of 39 wt.%).


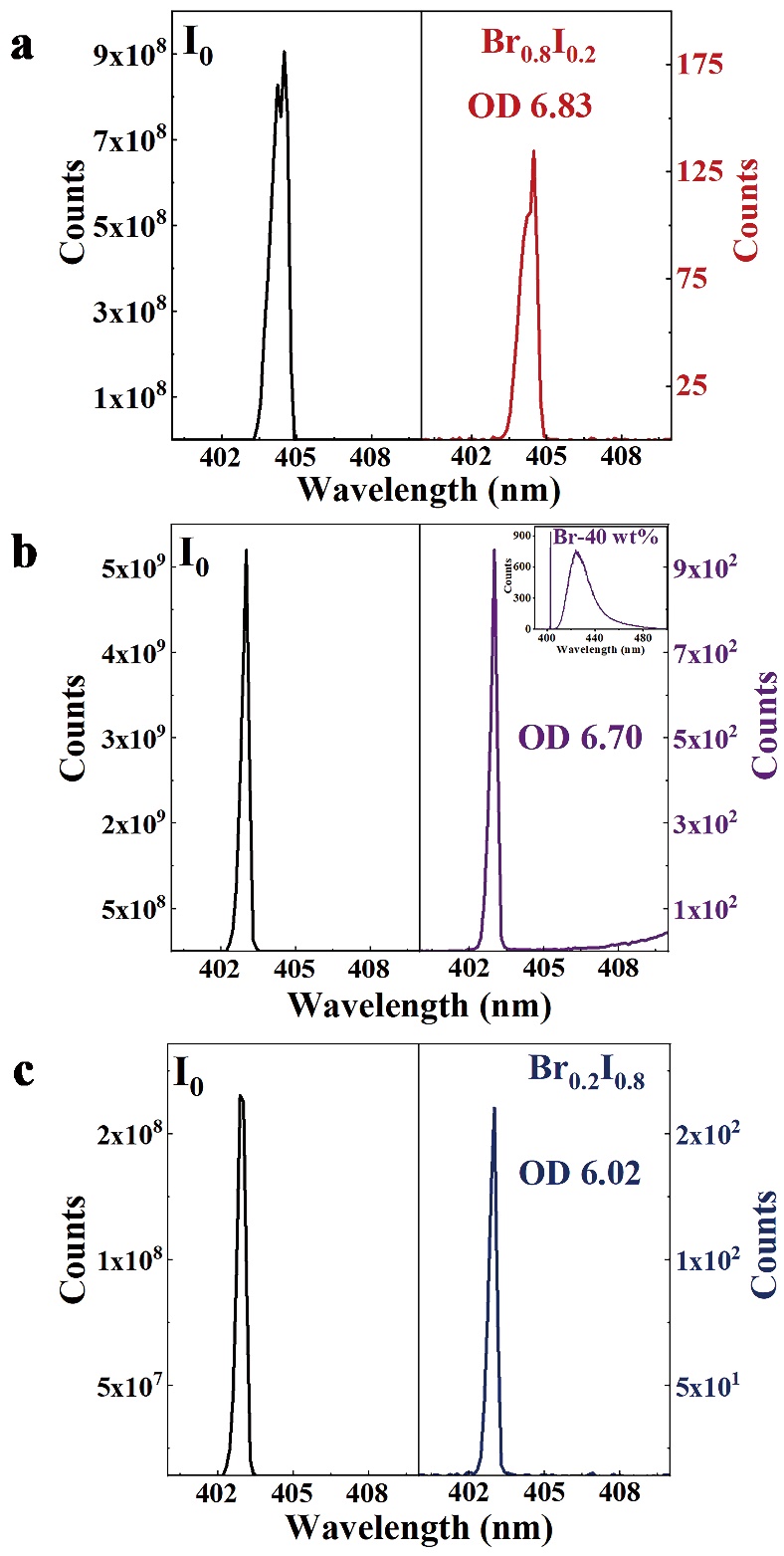


**Fig. S5. Size distribution and average diameter (D) of PQDFs based on a, MA_3_Bi_2_Cl_9_/PAN (D = 17.9 nm); b, MA_3_Bi_2_Cl_9_/PAN (D = 24.8 nm); c, MA_3_Bi_2_Cl_9_/PAN(D = 33.1 nm); and d, Cs_2_SnI_6_/PAN(D = 9.5 nm).**


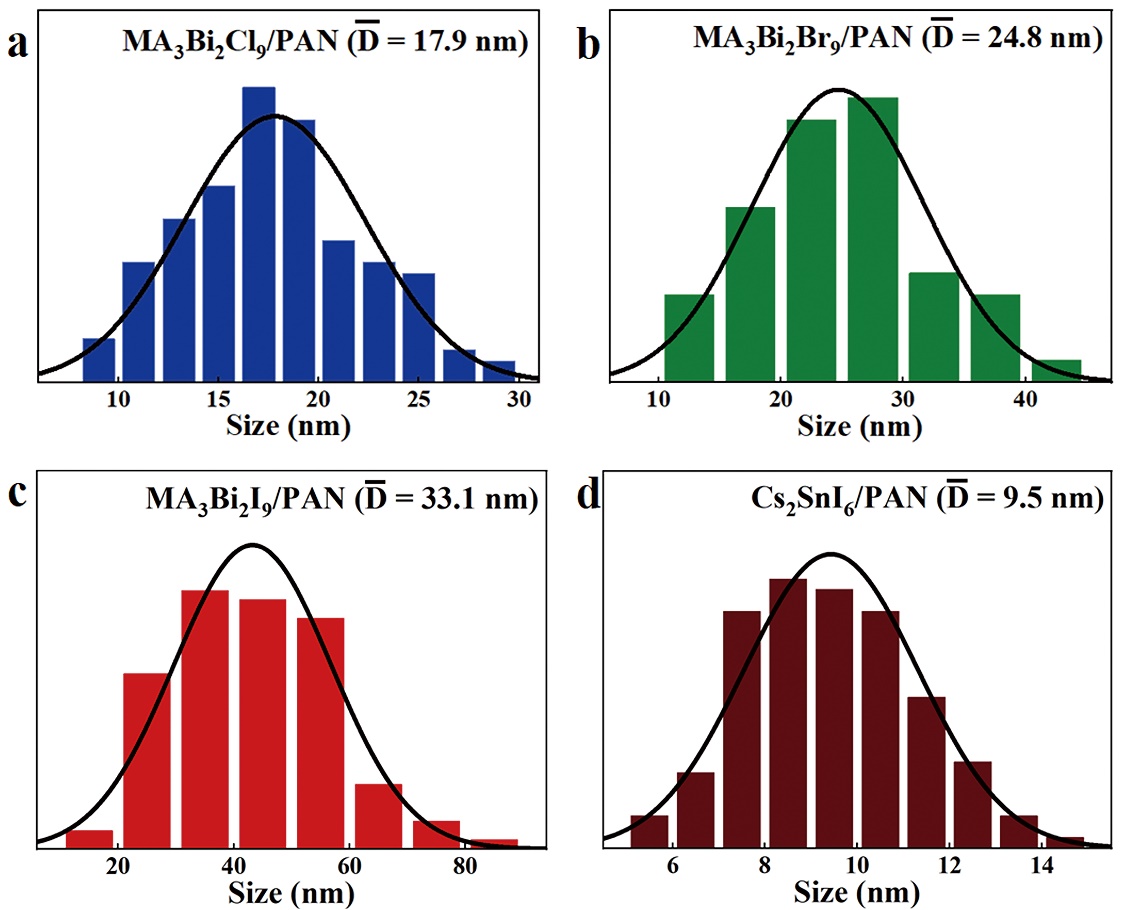


**Fig. S6. Transmittance spectra of PQDFs.** **a,** Transmittance spectra of MA_3_Bi_2_Cl_9_/PAN with a solid content of 5 wt.% and thickness of 20 μm. **b,** Transmittance spectra of filters for spectral resolution simulation.


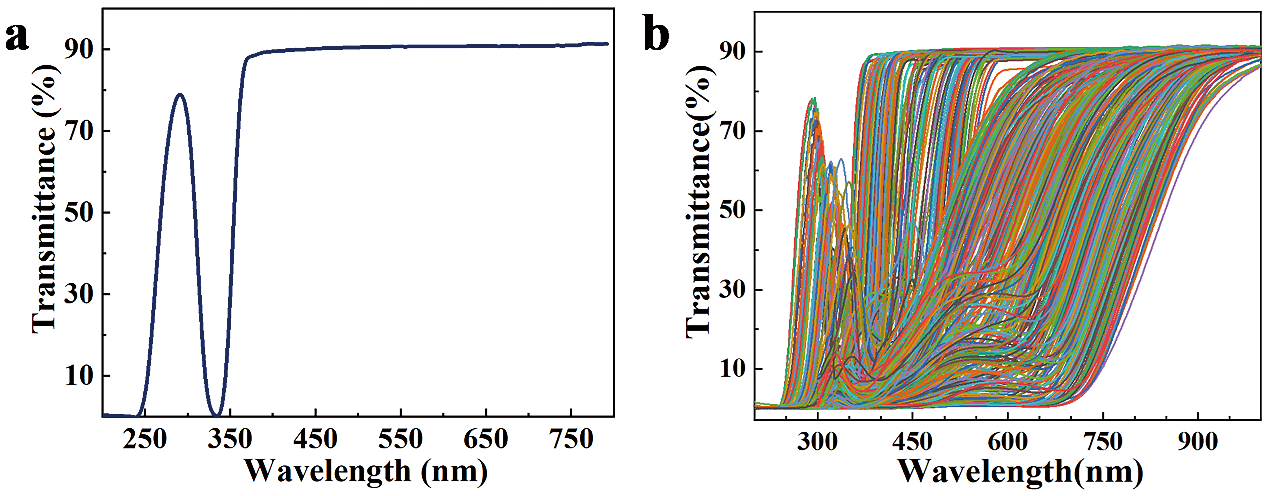


**Fig. S7. Experimental setup for the quantum dot spectral calibration.**


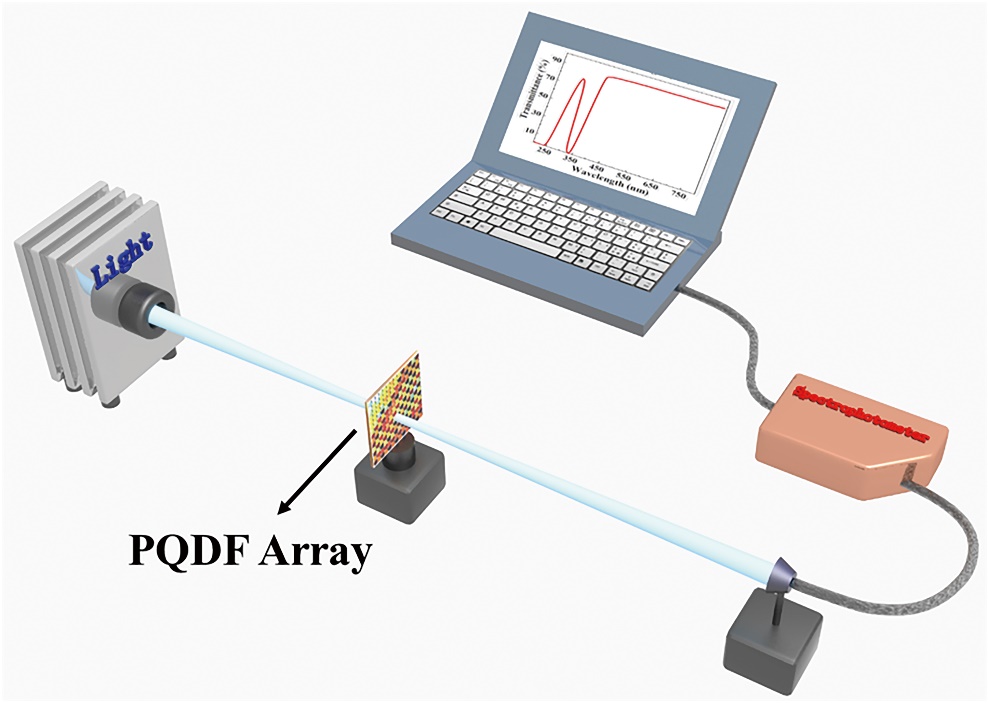


**Fig. S8. Transmittance spectra of filter array of PQDFs in the range from 400 nm to 950 nm.**


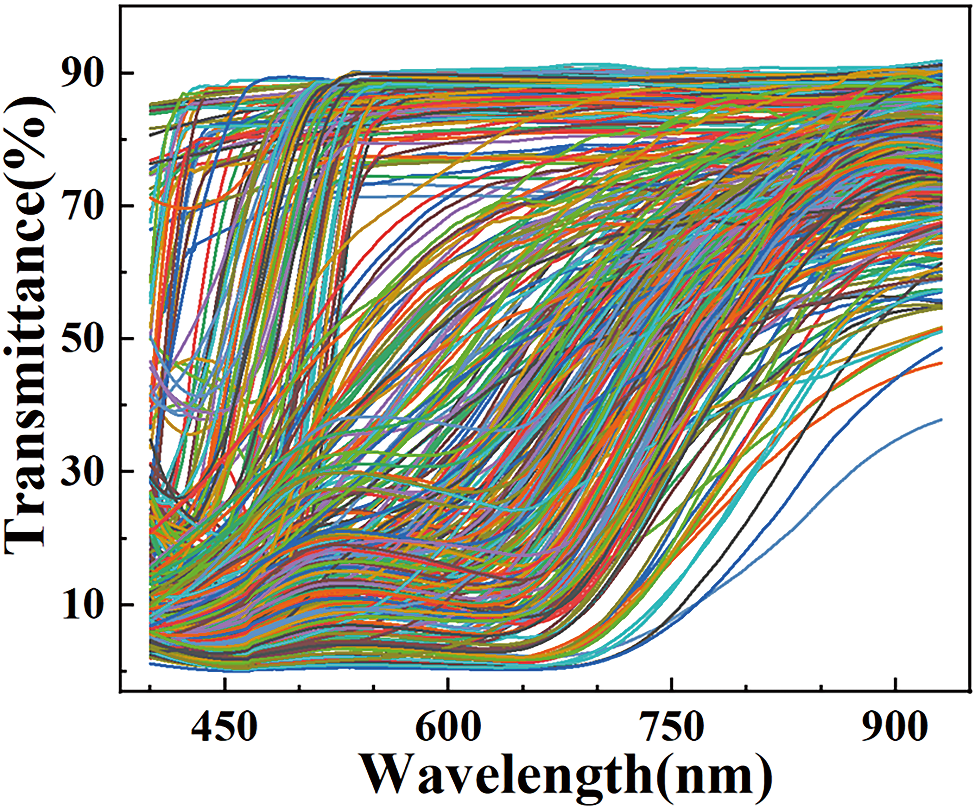

Supplement: Supplementary file 1 — Supplementary information [file 41377_2020_301_MOESM1_ESM.docx]
