# Supplementary material for: Broadband perovskite quantum dot spectrometer beyond human visual resolution
Source: Light Sci Appl. 2020 Apr 29;9:73. doi: 10.1038/s41377-020-0301-4 (PMC7190644; doi:10.1038/s41377-020-0301-4)
Supplement: Supplementary file 2 — Confidential Certificate [file 41377_2020_301_MOESM2_ESM.pdf]

文章保密与发表审查单

承　诺　书

此文章不涉密且不存在造假、抄袭、一稿多投等学术不端行为，特此承诺。

第一（通讯）作者签字：钟海政

年　　月　　日

《Light: Science & Applications》编辑部：

朱晓秀、边丽蘅、傅毫、王岭雪、邹炳锁、戴琼海、张军、钟海政作者为你刊撰写的文章

（题目：*Broadband perovskite quantum dot spectrometer beyond human visual resolution*），经审查，未发现该文章存在涉密内容和造假、抄袭、一稿多投等学术不端现象。该文章一经录用，其数字化复制权、发行权、汇编权及信息网络传播权将转让予《Light: Science & Applications》编辑部。

钟海政

导师（课题负责人）签字　　通讯或第一作者的单位或单位保密机构盖章

北京理工大学保密办公室 保密审查专用章

2020 年 3 月 12 日　　　　2020 年 3 月 12 日
